# Improvement of primary care for patients with chronic heart failure: a pilot study

**DOI:** 10.1186/1472-6963-10-8

**Published:** 2010-01-08

**Authors:** Jan van Lieshout, Michel Wensing, Richard Grol

**Affiliations:** 1 Scientific Institute for Quality of Healthcare, Radboud University Nijmegen Medical Centre, P.O. Box 9101, 114 IQ healthcare, 6500 HB Nijmegen, The Netherlands

## Abstract

**Background:**

Many patients with chronic heart failure (CHF) receive treatment in primary care, but data have shown that the quality of care for these patients needs to be improved. We aimed to evaluate the impact and feasibility of a programme for improving primary care for patients with CHF.

**Methods:**

An observational study was performed in 19 general practices in the south-eastern part of the Netherlands, evaluation involving 15 general practitioners and 77 CHF patients. The programme for improvement comprised educational and organizational components and was delivered by a trained practice visitor to the practices. The evaluation was based on case registration forms completed by health professionals and telephone interviews.

**Results:**

Management relating to diet and physical exercise seemed to have improved as eight patients were referred to dieticians and five to physiotherapists. The seasonal influenza vaccination rate increased from 94% to 97% (75/77). No impact on smoking was observed. Pharmaceutical treatment was adjusted according to guideline recommendations in 12% of the patients (9/77); 7 patients started recommended medication and 2 patients received dosage adjustments. General practitioners perceived the programme to be feasible. Clinical task delegation to nurses and assistants increased in some practices, but collaboration with other healthcare providers remained limited.

**Conclusions:**

The improvement programme proved to have moderate impact on patient care. Its effectiveness should be tested in a larger rigorous evaluation study using modifications based on the pilot experiences.

## Background

Heart failure is a chronic disease, which has high prevalence, high burden for patients, high mortality, and high costs of healthcare. The prevalence of chronic heart failure (CHF) in the western world is 1-2% in the general population and 10% or higher in the age group of 85 years and older [1,2]. Hospitalization with CHF as main diagnosis occurred in 2004 in 1.5 per 1.000 men and women, and mortality rates in heart failure patients are - with about 50% in 5 years - markedly higher compared to their age group without heart failure [2,3].

International clinical guidelines for the management of CHF provide comparable recommendations on diagnosis, treatment and lifestyle advice [4,5]. The recommended pharmaceutical treatment is complex and studies have reported suboptimal adherence to recommended drug treatment [6-11]. Providing education and counselling on lifestyle issues is recommended, despite variable results. Many patients with CHF receive treatment in outpatient hospital clinics settings and various programmes have been developed to improve the treatment in these settings [12]. However, in countries with a strong primary care system, a large group of patients with CHF receives treatment in primary care. This poses specific challenges, as primary care physicians often work in office-based practices, which may be less equipped to provide structured care for CHF than specialised hospital departments. Therefore, we developed a programme to improve primary care for CHF, comprising educational and organizational components. The programme included educational materials for physicians to instruct them on the recently updated recommendations on treatment, an algorithm which summarized the recommendations, and educational materials for patients. The organizational components comprised advice on organisational development of the practice, particularly focused on delegation of clinical tasks to practice assistants and nurses, which was delivered by a trained practice visitor. The underlying expectation was that this multifaceted programme would effectively improve patient care [13]. The aim of our study was to examine the impact on patient care and the feasibility of the programme.

## Methods

### Design

The study had a prospective observational design, with a six-month follow up period. A mixed methods approach was used, including both qualitative and quantitative data-collection. Quantitative data included changes in lifestyle advice and medication during the study period. The medical ethical committee (CMO Regio Arnhem - Nijmegen) assessed the study proposal and judged that the study could be conducted without its approval.

### Participants

The study population consisted of general practitioners (GPs) recruited in two regions in the southeastern part of the Netherlands. GPs were randomly selected from a national list and then approached for this study. Participating GPs were asked to include patients with CHF from their practice of whom the GPs considered themselves to be the physician taking care of the treatment of this condition in the patient. On average, a GP in the Netherlands has 25 patients listed with heart failure and about half of them receive their CHF treatment in primary care [14,15].

### Improvement programme

The programme comprised educational and organizational components, targeted at physicians, nurses and assistants, and patients. The educational component included a written summary of the non-pharmaceutical and pharmaceutical treatment recommendations. The information on non-pharmaceutical treatment concerned physical exercise, diet, smoking cessation, influenza vaccination, and materials to support advice to patients. The information on pharmaceutical treatment concerned the different drug groups advised: diuretics, ACE inhibitors or ARBs, beta blocking agents, aldosteron blocking agent, and digoxin. The contents were based on the prevailing Dutch College of General Practitioners' (DCGP) practice guideline on heart failure, revised and issued in 2005 with new recommendations on the use of ACE-inhibitors or angiotensin receptor blockers (ARBs), and on beta-blocker treatment [16].

In addition, we developed a form that summarized the various therapeutical options. We offered two-page case registration forms on which the GP could register the treatment at the start of the programme for each individual patient. The first page of the form guided the GP, nurse or practice assistant through the lifestyle issues and recommended giving advice when appropriate. The second page offered a stepwise overview through the drug treatment options. Possible changes in treatment could be noted. When a patient did not receive a certain indicated treatment option a question about the reason followed.

The organizational component of the programme comprised tailored advice on the practice organisation. Practices were encouraged to come to agreement about collaboration with other care providers and on the delegation of clinical tasks to nurses within the practice. To support this, a model for cooperation and task delegation was presented. We suggested that nurses and assistants could give non-pharmaceutical advice and have follow up contacts for monitoring side effects, blood pressure and other relevant clinical parameters during drug treatment titration. Also, in a stable phase periodical control visits could be delegated. A scheme was provided, offering an overview of tasks that could be delegated and offering advised moments of consultation with the GP. Furthermore, practices were encouraged to build a structured organization of the care for heart failure patients, which included regularly planned contacts with heart failure patients to evaluate their treatment, on a 3-months basis in a stable phase and with 2 to 4 weeks intervals during medication adjustments. Another organisational aspect of the improvement programme was that practices were encouraged to establish collaboration with physiotherapists and dieticians.

All materials were included in a package: the educational material, supportive materials, information on organisation, a scheme for task delegation, and the guiding patient information form. A trained practice visitor visited all practices. This practice visitor, a former practice assistant, was an experienced practice consultant trained in data collection and supporting practices in organizational changes. She clarified the project, introduced the folder, and checked if practices were able to select patients with heart failure appropriately. Six randomly assigned practices were offered three extra visits during the six-month period to study whether just introducing the material would be a feasible method and whether follow-ups were considered useful by the participating GPs. All other practices received one telephone call after four weeks to check whether they managed to fill in the patient registration forms. In follow up visits practices were encouraged to make organisational adjustments and to use the patient registration forms. Throughout the project period a GP with specific knowledge of treatment of CHF was available for questions raised by the participating GPs.

### Measures

Impact on patient care was assessed in terms of changes in drug treatment and life style advice given during the pilot period. This was based on the patient registration forms, which were completed by the GP, nurse or practice assistant for each included patient. The measures concerned treatment at the start of the programme, information on non-pharmaceutical treatment and medication changes with respect to heart failure during the programme period of 6 months. Practices were asked to send in anonymized duplicates of all patient registration forms at the end of the project period.

The feasibility of the programme was assessed in terms of GP reported experiences, using data from telephone interviews at the end of the project period. Our semi-structured questions focused on changes in collaboration with other care providers and task delegation within the practice, on time spent by GPs for our programme, and on the GPs views on the various components of the programme.

### Data-analysis

Results were categorized and presented descriptively.

### Ethical approval

The medical ethical committee (CMO Regio Arnhem - Nijmegen) assessed the study proposal and judged an approval was not necessary.

## Results

### Sample

In total 654 GPs were informed about our project by letter, of which 22 GPs agreed to participate in the study. Three GPs dropped out because of lack of time. Thirteen practices were visited just once at the project start. Six practices were visited three times; after the third visit they were offered another contact either by telephone or a visit. They all decided an extra visit was not necessary. When planning the telephone evaluations 4 GPs, all visited once, declared that they did not work with the materials at all. Therefore they did not participate in the evaluation. So, finally, 15 GPs participated in the evaluation; 10 of them sent in 77 patient registration forms. All GPs were able to select patients using ICPC codes. Table 1 summarizes these data. The study period was from October 2007 until April 2008.

**Table 1:** Flow of included practices and patients.

	Group 1 (three visits)	Group 2 (one visit)	Total
Included practices	7	15	22

Refused to participate	1	2	3

Participating practices	6	13	19

Number of practices participating in the evaluation	6	9	15

Number of practices returning patient information forms	5	5	10

Number of patient registration forms	35 (4-10)	42 (4-10)	77 (4-10)

Table 2 shows data on the 19 participating GPs and their practices and Table 3 shows demographic data of the patients included. All but one participating practices had one or more practice nurses. In seven practices these nurses already had clinical tasks in CHF care. Most GPs judged the DCGP's clinical practice guideline programme in general and the guideline on CHF in particular, positively. Involvement of a dietician or a physiotherapist in the care for heart failure patients was formalized in three and two practices respectively. Eleven GPs reported formal involvement of a cardiologist, one reported formal involvement of a heart failure nurse and one involved a heart failure outpatient clinic.

**Table 2:** Baseline characteristics of the general practices (total n = 19).

	N	%
Number of GPs in the practice:		
Solo practice	12	63
Duo practice	5	26
Group practice	2	11

Number of practices with practice support	18	95

Practice supportive staff member with tasks in heart failure	7	37

Number of practices accreditated	5	26

Agreement with contents of heart failure practice guideline	16	84

Positive attitude towards practice guidelines in general	17	89

Formalized cooperation with dietician	3	16

Formalized cooperation with physiotherapist	2	11

Formalized cooperation with cardiologist	11	58

Formalized cooperation with others (heart failure nurse, heart failure outpatient clinic)	2	11

**Table 3:** Patient characteristics at baseline (n = 77).

	n = 77	%
Sex		
Male	32	42
Female	44	58

Mean age (youngest and oldest)	78 sd 10,3 (44-92)	
Years since diagnosis on average	6	

NYHA class		

I	16	23

II	29	41

III	19	27

IV	7	10

Smoking		

Yes	9	13

No	60	87

Causes of heart failure (more than one possible):		

- Coronary heart disease	36	47

- Hypertension	44	57

- Others (e.g. rhythm disorders)	19	25

Co morbidity		

- Myocardial infarction in medical history	16	21

- Angina pectoris	25	32

- CABG or PCI in medical history	8	10

- Hypertension	34	44

- Asthma	3	4

- COPD	13	17

- Diabetes mellitus	24	31

- Hypothyroid disease	3	4

- Hyperthyroid disease	3	4

- Rhythm disorders, e.g. atrial fibrillation	11	14

- Others	52	68

Percentages are from the forms with data on the question, ignoring forms with missing data.

The patient sample included slightly more females (58%) than males, and the mean age was 78. About one third of the patients (37%) was in NYHA class III or IV, indicating more severe CHF. The GPs reported hypertension as one of the causes of heart failure in 57% of the patients and coronary heart disease in 47%.

The expert GP had telephone contacts with 3 GPs and email contacts with another 3 practices. One of the participating GPs had questions considering treating octogenarians; other issues were organizational.

### Impact

Table 4 shows the impact on lifestyle advice and drug treatment. Eight out of the 17 patients who received (renewed) dietary advice eight were referred to a dietician. Also, out of 28 patients who were advised on physical activity 5 patients were referred for physical activity therapy. Seasonal influenza vaccination grade was already high (94%, 72/77), but during the winter in the project period another three more patients were vaccinated. Of the patients registered as smokers 4 were (again) advised to stop smoking. None of them actually quitted smoking.

**Table 4:** Medical treatment at baseline and changes during the 6-month programme period.

	Baseline scores		Patients with changes since baseline	
	**n = 77**	**%**	**n = 77**	**%**

Non pharmaceutical treatment:				

Stop smoking advise given	6	8	4	5

Dietary advice given	39	51	17	22

Advise on physical activity given	18	23	28	36

Influenza vaccination provided	72	94	3	4

				

Drug treatment:				

Diuretics (thiazide or lisdiuretic) prescribed	63	82	4	5

ACE-inhibitor or ARB prescribed	58	75	3^1^	4

Beta blocker prescribed	43	56	0^1^	0

Spironolacton prescribed	15	19	0	0

Digoxin	5	6	0	0

^1 ^One patient on medication already had dosage adjustment according to the recommendations

In 4 patients their GP started a diuretic and in 3 patients an ACE inhibitor or ARB in accordance with the recommendations. Furthermore, in one patient ACE inhibitor dosage was optimized. Although just over half of the patients were treated with a beta-blocker, the GPs did not start beta-blocker treatment with any patient. On the patient registration, all patients with beta-blockers indicated but not prescribed were said to have a contra indication. In one patient the beta-blocker dosage was optimized. In total, medication was adjusted in 9 patients (12%).

### Feasibility

All elements of the programme were evaluated positively by the vast majority of the 15 GPs involved in the evaluation. Of them, 14 had positive judgements about both the written materials and the practice visits, and 12 about the patient registration forms. Thirteen GPs would advise colleagues to participate in this heart failure programme and would participate in a programme like this again. The mean time investment for the GP was 2.5 hours, ranging from 0.5 to 5 hours.

Six GPs made organisational changes in the management of chronic heart failure care. Only a few GPs considered cooperation with other disciplines in the care improved: three GPs with a dietician, one with a physiotherapist, and one with a cardiologist. Nine GPs considered care for heart failure patients in their practice as improved.

In the group with repeated visits all practices were satisfied in their need for support after 3 visits although another follow up visit was offered. One GP thought the support was more than necessary. In the group with just one practice visit 4 GPs would have welcomed more support; the other 5 GPs were satisfied with just one visit.

## Discussion

### Main conclusions

The programme to improve primary care for patients with CHF proved to be feasible and to be associated with clinically relevant improvements in at least some patients, particularly regarding lifestyle advice. All components of the programme - the written material including the patient registration forms and the practice visits by the outreach visitor - were positively assessed. There was no clear optimum number of visits by the practice visitor (one or three). Obviously, the need for visits will differ between practices. In a number of practices one practice visit did not suffice, as in the group of 13 practices that were visited once only 9 GPs finally worked with the materials provided, and 4 of these 9 GPs considered support with one visit insufficient.

### Interpretation

Especially in specialist medical care, there have been several initiatives for improving healthcare for patients with CHF. Many of these programmes include more frequent contacts with the patients by specialized heart failure nurses than usual care provides. The outcomes of these heart failure programmes were not consistent [17-19].

A limited number of randomised controlled trials evaluated improvement programmes for the treatment of heart failure in primary care [20-24]. Interventions consisted of nurse-led contacts, computer based treatment suggestions, practice guideline recommendations and disease management programmes, for instance. Again, outcomes were not consistent, with significant differences in some endpoints defined, but not in all. Therefore, also in primary care, further effort is necessary to develop effective improvement programmes.

Our programme aimed to achieve changes by reducing various types of barriers for change, the complexity of the prevailing practice guideline with recently changed treatment recommendations being one. Therefore we provided educational interventions. Secondly, in CHF care there was no structured approach as there is in diabetes care. We proposed organizational interventions, which were tailored to the practice. Although the impact of tailoring and combining interventions has not been found to be consistently more effective than single interventions [13], many experts have proposed that these two aspects contribute to the effectiveness of programmes for improving healthcare [25]. A problem, however, is that tailoring interventions to organizational barriers is difficult [26].

Our patient sample seemed to represent the population quite well, considering sex, age, pathology underlying CHF, and co-morbidity [8,11]. The rather high percentage of NYHA class III or IV (37%) might be unexpected in primary care. Yet, especially in the group of old and severely ill, patients usually do not visit the outpatient clinic and are treated in primary care with home visits.

Several studies showed that pharmaceutical treatment of heart failure patients in general practices is not optimal. Forty to 78% of the heart failure patients were treated with an ACE-inhibitor or ARB [6-11]. In our sample 82% of the patients were using an ACE-inhibitor or ARB. The use of beta-blockers in previous studies varied from 7 to 32% [8,10,11]. In our study 56% of the patients used a beta-blocking agent. Our relatively high percentages may indicate a selection of practices or patients, but may also reflect the fact that our data were collected more recently. Revised practice guideline recommendations for Dutch GPs have been published in 2005. Though treatment was good already there was still room for improvement, and the percentage of patients who received improved care reflects percentages found in previous studies of implementation interventions [27].

We did not find much change in formalized interdisciplinary cooperation with other care providers. Our programme did not contain supportive materials for improvement of interdisciplinary cooperation (for instance with dieticians). This clearly has to be addressed in further development of the programme, following for instance a comprehensive tool with a list of tasks to make interdisciplinary cooperation agreement made by The Dutch Heart Foundation [28].

### Strengths and weaknesses

We carefully developed a programme for improving primary care for CHF patients, and tested this in real practice. This is an important step in the development of effective interventions, which is often overlooked. A serious limitation of our study is the practice sample and the patient sample size. The sample of practices is non-random due to the low response rate, and the patient sample is small (77 patients included) because only 10 GPs sent back the registration forms. Generalizing results beyond the practices and patients studied is problematic, but for the purpose of this study this was regarded as acceptable.

The strength of our programme is the fact that it is multifaceted, addressing organizational and educational aspects with a variety of materials and visits. Especially, the patient registration form with an algorithm was guiding and very helpful.

### Implications for research, policy and practice

Some aspects of the programme can be improved, particularly regarding collaboration with other care providers, making use of existing materials. Our programme was entirely focussed on treatment; medical education on the diagnostic work up could serve as a starting point for a kick off of the programme. The programme held many elements. A barrier analysis in each participating practice could identify the need for specific elements and form the basis of a tailored made intervention. Within one region other disciplines could be invited to participate, reinforcing treatment in primary care in general. Also, cooperation with cardiologists could be enforced with local agreement meetings on diagnostic procedures, referral and referring back to primary care, and shared care. In such a new approach the programme should be evaluated again. Then, the major challenge is to make the programme available for larger groups of primary care practices. The programme could gain strength with a more regional approach, which would give opportunities for cooperation especially when functioning regional organisations or structures are available.

## Conclusion

A feasible and promising programme for improving primary care for patients with CHF is now available. Its effectiveness needs to be tested in a larger study after making programme adjustments taking advantage of the lessons learned in this pilot study. Further implementation of the programme could be organized on a regional level. Extra attention should be paid to cooperation with other disciplines and to making use of existing structures within these regions.

## Competing interests

The authors declare that they have no competing interests.

## Authors' contributions

JvL contributed to the conception and design of the study, to the development of the implementation materials, to the realization of the study, the data collection and analysis, and drafted and revised the manuscript. MW contributed to the conception and design of the study, to the development of the implementation materials, and revised the manuscript critically for important intellectual content. RG revised the manuscript critically for important intellectual content. All authors read and approved the final manuscript.

## Pre-publication history

The pre-publication history for this paper can be accessed here:

http://www.biomedcentral.com/1472-6963/10/8/prepub
